# Crystal structure of di­chlorido­(2,2′:6′,2′′-terpyridine-κ^3^ *N*,*N*′,*N*′′)zinc: a redeter­min­ation

**DOI:** 10.1107/S1600536814023605

**Published:** 2014-10-31

**Authors:** Cheng-Cheng Kong, Jia-Zheng Zhou, Jian-Hua Yu, Sheng-Li Li

**Affiliations:** a Deparment of Chemistry, Anhui University, Hefei 230039, Peoples Republic of China, Key Laboratory of Functional Inorganic Materials, Chemistry, Hefei 230039, People’s Republic of China

**Keywords:** crystal structure, redetermination, 2,2′:6′,2′′-terpyridine, zinc complex, π–π inter­actions

## Abstract

The crystal structure of the title compound, [ZnCl_2_(C_15_H_11_N_3_)], was redetermined based on modern CCD data. In comparison with the previous determination from photographic film data [Corbridge & Cox (1956 ▶). *J. Chem. Soc.* **159**, 594–603; Einstein & Penfold (1966 ▶). *Acta Cryst.* **20**, 924–926], all non-H atoms were refined with anisotropic displacement parameters, leading to a much higher precision in terms of bond lengths and angles [*e.g.* Zn—Cl = 2.2684 (8) and 2.2883 (11) compared to 2.25 (1) and 2.27 (1) Å]. In the title mol­ecule, the Zn^II^ atom is five-coordinated in a distorted square-pyramidal mode by two Cl atoms and by the three N atoms from the 2,2′:6′,2′′-terpyridine ligand. The latter is not planar and shows dihedral angles between the least-squares planes of the central pyridine ring and the terminal rings of 3.18 (8) and 6.36 (9)°. The mol­ecules in the crystal structure pack with π–π inter­actions [centroid–centroid distance = 3.655 (2) Å] between pyridine rings of neighbouring terpyridine moieties. These, together with inter­molecular C—H⋯Cl inter­actions, stablize the three-dimensional structure.

## Related literature   

The title compound is dimorphic, with one polymorph (form I) crystallizing in space group No. 15, and the second polymorph (type II) crystallizing in space group No. 14 (Corbridge & Cox, 1956 ▶). The crystal structure of the title compound was originally determined by Corbridge & Cox (1956 ▶) from photographic data (final *R* value = 0.24) and was later re-refined by Einstein & Penfold (1966 ▶) based on the original intensity data but using more advanced least-squares procedures (*R* = 0.14). In both reports, the setting in *P*2_1_/*a* of space group No. 14 was used. For background to terpyridine-based materials, see: Fermi *et al.* (2014 ▶); Song *et al.* (2014 ▶). For the biocompatibility of zinc compounds, see: Gao *et al.* (2009 ▶).
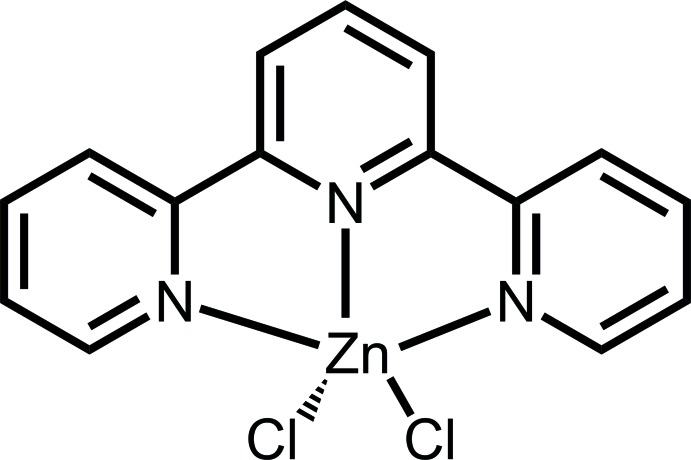


## Experimental   

### Crystal data  

[ZnCl_2_(C_15_H_11_N_3_)]
*M* *_r_* = 369.54
Monoclinic, 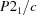

*a* = 10.950 (5) Å
*b* = 8.250 (5) Å
*c* = 16.216 (5) Å
β = 93.911 (5)°
*V* = 1461.5 (12) Å^3^
*Z* = 4
Mo *K*α radiation
μ = 2.04 mm^−1^
*T* = 298 K
0.30 × 0.20 × 0.20 mm

### Data collection  

Bruker SMART CCD diffractometer
Absorption correction: multi-scan (*SADABS*; Bruker, 2002 ▶) *T*_min_ = 0.580, *T*_max_ = 0.686
9990 measured reflections
2564 independent reflections
2404 reflections with *I* > 2σ(*I*)
*R*_int_ = 0.017

### Refinement  

*R*[*F* ^2^ > 2σ(*F* ^2^)] = 0.020
*wR*(*F* ^2^) = 0.056
*S* = 1.05
2564 reflections
190 parameters
H-atom parameters constrained
Δρ_max_ = 0.24 e Å^−3^
Δρ_min_ = −0.24 e Å^−3^

### 

Data collection: *SMART* (Bruker, 2002 ▶); cell refinement: *SAINT* (Bruker, 2002 ▶); data reduction: *SAINT*; program(s) used to solve structure: *SHELXS97* (Sheldrick, 2008 ▶); program(s) used to refine structure: *SHELXL97* (Sheldrick, 2008 ▶); molecular graphics: *SHELXTL* (Sheldrick, 2008 ▶) and *DIAMOND* (Brandenburg, 2006 ▶); software used to prepare material for publication: *SHELXTL* (Sheldrick, 2008 ▶).

## Supplementary Material

Crystal structure: contains datablock(s) I, Global. DOI: 10.1107/S1600536814023605/wm5082sup1.cif

Structure factors: contains datablock(s) I. DOI: 10.1107/S1600536814023605/wm5082Isup2.hkl

**Fig. 1.**
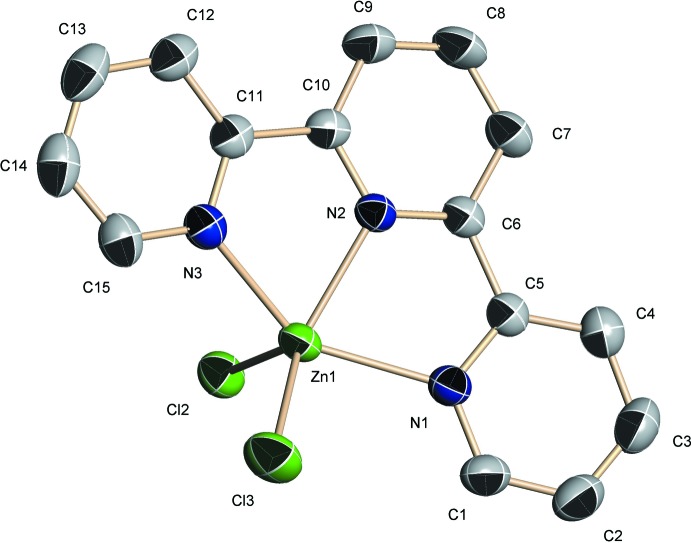
The molecular structure of (I), with the atom-numbering scheme. Displacement ellipsoids are drawn at the 50% probability level.

. DOI: 10.1107/S1600536814023605/wm5082fig1.tif
The mol­ecular structure of (I), with the atom-numbering scheme. Displacement ellipsoids are drawn at the 50% probability level.

**Fig. 2.**
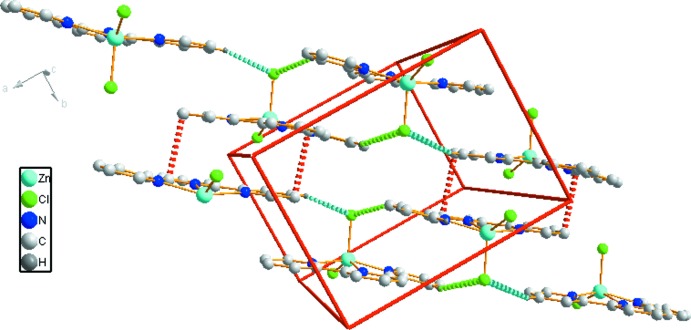
The arrangement of the molecules in the crystal structure of (I), showing π—π interactions (dashed red lines) and C—H···Cl hydrogen bonds (dashed green and turquoise lines).

. DOI: 10.1107/S1600536814023605/wm5082fig2.tif
The arrangement of the mol­ecules in the crystal structure of (I), showing π—π inter­actions (dashed red lines) and C—H⋯Cl hydrogen bonds (dashed green and turquoise lines).

**Fig. 3.**
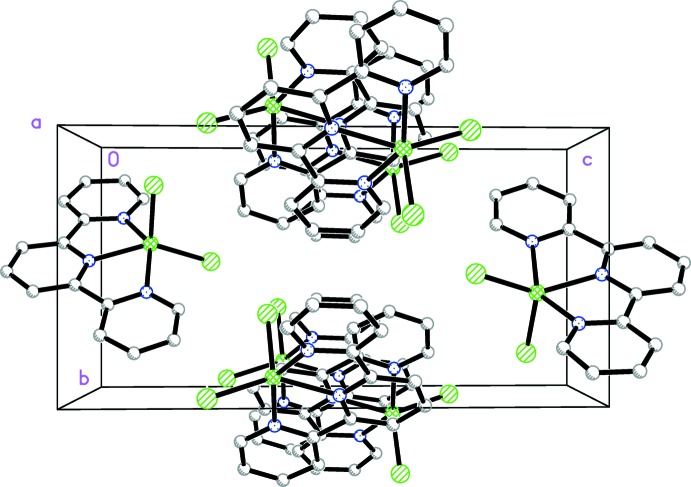
Packing diagram of (I). All H atoms have been omitted for clarity.

. DOI: 10.1107/S1600536814023605/wm5082fig3.tif
Packing diagram of (I). All H atoms have been omitted for clarity.

CCDC reference: 1029855

Additional supporting information:  crystallographic information; 3D view; checkCIF report

## Figures and Tables

**Table 1:** Hydrogen-bond geometry (, )

*D*H*A*	*D*H	H*A*	*D* *A*	*D*H*A*
C4H4Cl2^i^	0.93	2.68	3.518(2)	151
C13H13Cl2^ii^	0.93	2.81	3.686(2)	158

Symmetry codes: (i) 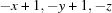; (ii) 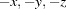.

## supplementary crystallographic information

### S1. Experimental

A solution of 2,2':6',2''-terpyridine (0.23 g, 1 mmol) in acetonitrile (20 ml) was mixed with a solution of zinc chloride (0.14 g, 1 mmol) in methanol (5 ml) and refluxed for 4 h. The reaction mixture was the cooled to room temperature and filtered into a large test tube. Colorless crystals were obtained at room temperature after two weeks. Yield: 75%.

### S2. Refinement

All hydrogen atoms were placed in geometrically idealized positions and constrained to ride on their parent atoms, with C—H = 0.93 Å and *U*_iso_(H) = 1.2*U*_eq_(C).

### Crystal data

**Table:**

[ZnCl_2_(C_15_H_11_N_3_)]	*F*(000) = 744
*M**_r_* = 369.54	*D*_x_ = 1.679 Mg m^−^^3^
Monoclinic, *P*2_1_/*c*	Mo *K*α radiation, λ = 0.71069 Å
Hall symbol: -P 2ybc	Cell parameters from 6593 reflections
*a* = 10.950 (5) Å	θ = 2.8–26.9°
*b* = 8.250 (5) Å	µ = 2.04 mm^−^^1^
*c* = 16.216 (5) Å	*T* = 298 K
β = 93.911 (5)°	Block, colorless
*V* = 1461.5 (12) Å^3^	0.30 × 0.20 × 0.20 mm
*Z* = 4	

### Data collection

**Table:**

Bruker SMART CCD diffractometer	2564 independent reflections
Radiation source: fine-focus sealed tube	2404 reflections with *I* > 2σ(*I*)
Graphite monochromator	*R*_int_ = 0.017
φ and ω scans	θ_max_ = 25.0°, θ_min_ = 1.9°
Absorption correction: multi-scan (*SADABS*; Bruker, 2002)	*h* = −13→12
*T*_min_ = 0.580, *T*_max_ = 0.686	*k* = −9→9
9990 measured reflections	*l* = −18→19

### Refinement

**Table:**

Refinement on *F*^2^	Primary atom site location: structure-invariant direct methods
Least-squares matrix: full	Secondary atom site location: difference Fourier map
*R*[*F*^2^ > 2σ(*F*^2^)] = 0.020	Hydrogen site location: inferred from neighbouring sites
*wR*(*F*^2^) = 0.056	H-atom parameters constrained
*S* = 1.05	*w* = 1/[σ^2^(*F*_o_^2^) + (0.0322*P*)^2^ + 0.4083*P*] where *P* = (*F*_o_^2^ + 2*F*_c_^2^)/3
2564 reflections	(Δ/σ)_max_ < 0.001
190 parameters	Δρ_max_ = 0.24 e Å^−^^3^
0 restraints	Δρ_min_ = −0.24 e Å^−^^3^

### Special details

**Table:**

Geometry. All e.s.d.'s (except the e.s.d. in the dihedral angle between two l.s. planes) are estimated using the full covariance matrix. The cell e.s.d.'s are taken into account individually in the estimation of e.s.d.'s in distances, angles and torsion angles; correlations between e.s.d.'s in cell parameters are only used when they are defined by crystal symmetry. An approximate (isotropic) treatment of cell e.s.d.'s is used for estimating e.s.d.'s involving l.s. planes.
Refinement. Refinement of *F*^2^ against ALL reflections. The weighted *R*-factor *wR* and goodness of fit *S* are based on *F*^2^, conventional *R*-factors *R* are based on *F*, with *F* set to zero for negative *F*^2^. The threshold expression of *F*^2^ > σ(*F*^2^) is used only for calculating *R*-factors(gt) *etc*. and is not relevant to the choice of reflections for refinement. *R*-factors based on *F*^2^ are statistically about twice as large as those based on *F*, and *R*- factors based on ALL data will be even larger.

### Fractional atomic coordinates and isotropic or equivalent isotropic displacement parameters (Å^2^)

**Table:**

	*x*	*y*	*z*	*U*_iso_*/*U*_eq_	
Zn1	0.217983 (18)	0.40487 (2)	0.117728 (11)	0.03587 (8)	
Cl2	0.33535 (4)	0.17571 (5)	0.13148 (3)	0.04477 (12)	
Cl3	0.13940 (5)	0.47642 (7)	0.23837 (3)	0.06000 (15)	
N1	0.35875 (14)	0.59791 (17)	0.11822 (9)	0.0384 (3)	
N2	0.22895 (12)	0.47354 (16)	−0.00704 (8)	0.0324 (3)	
N3	0.05709 (13)	0.29671 (17)	0.05158 (9)	0.0385 (3)	
C4	0.48010 (17)	0.7621 (2)	0.03495 (12)	0.0471 (4)	
H4	0.5007	0.7944	−0.0173	0.057*	
C5	0.39054 (15)	0.6462 (2)	0.04352 (10)	0.0353 (4)	
C10	0.15500 (16)	0.40050 (19)	−0.06497 (11)	0.0352 (4)	
C9	0.17350 (19)	0.4201 (2)	−0.14845 (11)	0.0462 (5)	
H9	0.1228	0.3690	−0.1888	0.055*	
C12	−0.03366 (17)	0.2236 (2)	−0.08134 (13)	0.0463 (4)	
H12	−0.0337	0.2290	−0.1386	0.056*	
C2	0.5060 (2)	0.7802 (2)	0.18111 (14)	0.0564 (5)	
H2	0.5441	0.8240	0.2291	0.068*	
C8	0.26887 (19)	0.5170 (3)	−0.16981 (11)	0.0497 (5)	
H8	0.2832	0.5304	−0.2253	0.060*	
C15	−0.02974 (17)	0.2127 (2)	0.08669 (13)	0.0459 (4)	
H15	−0.0286	0.2094	0.1441	0.055*	
C6	0.32011 (15)	0.56916 (19)	−0.02768 (10)	0.0336 (4)	
C11	0.05559 (15)	0.3024 (2)	−0.03164 (11)	0.0369 (4)	
C1	0.41594 (19)	0.6650 (2)	0.18517 (12)	0.0485 (5)	
H1	0.3938	0.6323	0.2370	0.058*	
C14	−0.12109 (18)	0.1307 (2)	0.04107 (15)	0.0522 (5)	
H14	−0.1802	0.0728	0.0673	0.063*	
C13	−0.12326 (18)	0.1362 (2)	−0.04368 (15)	0.0542 (5)	
H13	−0.1841	0.0819	−0.0756	0.065*	
C3	0.53854 (19)	0.8291 (3)	0.10520 (14)	0.0560 (5)	
H3	0.5994	0.9067	0.1008	0.067*	
C7	0.34327 (18)	0.5944 (2)	−0.11007 (11)	0.0430 (4)	
H7	0.4068	0.6613	−0.1244	0.052*	

### Atomic displacement parameters (Å^2^)

**Table:**

	*U*^11^	*U*^22^	*U*^33^	*U*^12^	*U*^13^	*U*^23^
Zn1	0.04271 (14)	0.03704 (13)	0.02811 (12)	0.00301 (8)	0.00414 (9)	0.00265 (7)
Cl2	0.0482 (3)	0.0413 (2)	0.0446 (3)	0.00621 (19)	0.00184 (19)	0.00566 (19)
Cl3	0.0765 (4)	0.0645 (3)	0.0410 (3)	0.0083 (3)	0.0181 (2)	−0.0013 (2)
N1	0.0457 (8)	0.0353 (8)	0.0339 (8)	0.0016 (6)	0.0007 (6)	0.0008 (6)
N2	0.0369 (7)	0.0307 (7)	0.0296 (7)	0.0045 (6)	0.0023 (6)	0.0007 (5)
N3	0.0390 (8)	0.0358 (8)	0.0410 (8)	0.0042 (6)	0.0045 (6)	0.0028 (6)
C4	0.0467 (11)	0.0434 (10)	0.0518 (11)	−0.0007 (8)	0.0073 (9)	0.0049 (8)
C5	0.0361 (9)	0.0319 (8)	0.0381 (9)	0.0066 (7)	0.0039 (7)	0.0012 (7)
C10	0.0390 (9)	0.0332 (9)	0.0330 (9)	0.0083 (7)	0.0005 (7)	−0.0021 (7)
C9	0.0546 (11)	0.0507 (11)	0.0328 (10)	0.0051 (9)	−0.0013 (8)	−0.0075 (8)
C12	0.0454 (10)	0.0379 (10)	0.0541 (11)	0.0058 (8)	−0.0071 (8)	−0.0063 (8)
C2	0.0657 (13)	0.0434 (11)	0.0571 (13)	−0.0012 (10)	−0.0170 (10)	−0.0062 (10)
C8	0.0623 (12)	0.0595 (12)	0.0284 (9)	0.0057 (10)	0.0120 (8)	0.0005 (8)
C15	0.0426 (10)	0.0410 (10)	0.0549 (11)	0.0047 (8)	0.0101 (8)	0.0073 (8)
C6	0.0359 (9)	0.0315 (8)	0.0335 (9)	0.0073 (7)	0.0045 (7)	0.0014 (7)
C11	0.0370 (9)	0.0304 (8)	0.0429 (10)	0.0077 (7)	−0.0015 (7)	−0.0017 (7)
C1	0.0646 (12)	0.0426 (11)	0.0370 (10)	0.0025 (9)	−0.0063 (9)	−0.0023 (8)
C14	0.0384 (10)	0.0358 (10)	0.0831 (16)	0.0036 (8)	0.0102 (10)	0.0056 (10)
C13	0.0402 (10)	0.0361 (10)	0.0844 (16)	0.0035 (8)	−0.0091 (10)	−0.0081 (10)
C3	0.0493 (11)	0.0444 (11)	0.0731 (15)	−0.0086 (9)	−0.0050 (10)	−0.0010 (10)
C7	0.0477 (11)	0.0461 (11)	0.0365 (10)	0.0045 (8)	0.0132 (8)	0.0037 (8)

### Geometric parameters (Å, º)

**Table:**

Zn1—N2	2.1123 (14)	C9—H9	0.9300
Zn1—N3	2.1893 (16)	C12—C11	1.386 (3)
Zn1—N1	2.2160 (17)	C12—C13	1.392 (3)
Zn1—Cl3	2.2684 (8)	C12—H12	0.9300
Zn1—Cl2	2.2883 (11)	C2—C3	1.365 (3)
N1—C1	1.336 (2)	C2—C1	1.375 (3)
N1—C5	1.343 (2)	C2—H2	0.9300
N2—C6	1.333 (2)	C8—C7	1.379 (3)
N2—C10	1.341 (2)	C8—H8	0.9300
N3—C15	1.335 (2)	C15—C14	1.380 (3)
N3—C11	1.349 (2)	C15—H15	0.9300
C4—C5	1.383 (3)	C6—C7	1.393 (2)
C4—C3	1.384 (3)	C1—H1	0.9300
C4—H4	0.9300	C14—C13	1.374 (3)
C5—C6	1.487 (2)	C14—H14	0.9300
C10—C9	1.392 (3)	C13—H13	0.9300
C10—C11	1.487 (2)	C3—H3	0.9300
C9—C8	1.378 (3)	C7—H7	0.9300
			
N2—Zn1—N3	74.72 (6)	C11—C12—H12	120.7
N2—Zn1—N1	74.08 (5)	C13—C12—H12	120.7
N3—Zn1—N1	146.20 (6)	C3—C2—C1	118.67 (19)
N2—Zn1—Cl3	143.70 (4)	C3—C2—H2	120.7
N3—Zn1—Cl3	100.88 (5)	C1—C2—H2	120.7
N1—Zn1—Cl3	96.58 (5)	C7—C8—C9	120.90 (17)
N2—Zn1—Cl2	104.28 (4)	C7—C8—H8	119.5
N3—Zn1—Cl2	97.98 (5)	C9—C8—H8	119.5
N1—Zn1—Cl2	101.98 (6)	N3—C15—C14	122.48 (19)
Cl3—Zn1—Cl2	111.99 (2)	N3—C15—H15	118.8
C1—N1—C5	118.30 (16)	C14—C15—H15	118.8
C1—N1—Zn1	126.03 (13)	N2—C6—C7	121.20 (16)
C5—N1—Zn1	115.64 (11)	N2—C6—C5	114.56 (14)
C6—N2—C10	121.12 (14)	C7—C6—C5	124.22 (16)
C6—N2—Zn1	119.46 (11)	N3—C11—C12	121.68 (17)
C10—N2—Zn1	118.66 (11)	N3—C11—C10	115.04 (15)
C15—N3—C11	118.97 (16)	C12—C11—C10	123.27 (17)
C15—N3—Zn1	125.12 (13)	N1—C1—C2	123.08 (19)
C11—N3—Zn1	115.48 (11)	N1—C1—H1	118.5
C5—C4—C3	119.01 (18)	C2—C1—H1	118.5
C5—C4—H4	120.5	C13—C14—C15	118.87 (19)
C3—C4—H4	120.5	C13—C14—H14	120.6
N1—C5—C4	121.64 (17)	C15—C14—H14	120.6
N1—C5—C6	114.90 (15)	C14—C13—C12	119.42 (19)
C4—C5—C6	123.44 (16)	C14—C13—H13	120.3
N2—C10—C9	120.56 (17)	C12—C13—H13	120.3
N2—C10—C11	114.32 (15)	C2—C3—C4	119.32 (19)
C9—C10—C11	125.12 (16)	C2—C3—H3	120.3
C8—C9—C10	118.35 (18)	C4—C3—H3	120.3
C8—C9—H9	120.8	C8—C7—C6	117.84 (17)
C10—C9—H9	120.8	C8—C7—H7	121.1
C11—C12—C13	118.6 (2)	C6—C7—H7	121.1
			
N2—Zn1—N1—C1	174.74 (16)	N2—C10—C9—C8	−0.5 (3)
N3—Zn1—N1—C1	151.48 (14)	C11—C10—C9—C8	179.05 (16)
Cl3—Zn1—N1—C1	30.57 (15)	C10—C9—C8—C7	−0.8 (3)
Cl2—Zn1—N1—C1	−83.62 (15)	C11—N3—C15—C14	0.2 (3)
N2—Zn1—N1—C5	−7.44 (11)	Zn1—N3—C15—C14	−171.91 (14)
N3—Zn1—N1—C5	−30.69 (17)	C10—N2—C6—C7	−1.2 (2)
Cl3—Zn1—N1—C5	−151.60 (11)	Zn1—N2—C6—C7	168.70 (12)
Cl2—Zn1—N1—C5	94.20 (11)	C10—N2—C6—C5	177.68 (14)
N3—Zn1—N2—C6	177.72 (13)	Zn1—N2—C6—C5	−12.46 (18)
N1—Zn1—N2—C6	10.88 (11)	N1—C5—C6—N2	5.3 (2)
Cl3—Zn1—N2—C6	90.10 (13)	C4—C5—C6—N2	−173.10 (16)
Cl2—Zn1—N2—C6	−87.75 (12)	N1—C5—C6—C7	−175.93 (16)
N3—Zn1—N2—C10	−12.17 (11)	C4—C5—C6—C7	5.7 (3)
N1—Zn1—N2—C10	−179.01 (13)	C15—N3—C11—C12	0.0 (2)
Cl3—Zn1—N2—C10	−99.79 (13)	Zn1—N3—C11—C12	172.94 (13)
Cl2—Zn1—N2—C10	82.36 (12)	C15—N3—C11—C10	179.28 (14)
N2—Zn1—N3—C15	−177.15 (15)	Zn1—N3—C11—C10	−7.83 (18)
N1—Zn1—N3—C15	−153.97 (13)	C13—C12—C11—N3	−0.2 (3)
Cl3—Zn1—N3—C15	−34.18 (14)	C13—C12—C11—C10	−179.42 (16)
Cl2—Zn1—N3—C15	80.15 (14)	N2—C10—C11—N3	−2.3 (2)
N2—Zn1—N3—C11	10.46 (11)	C9—C10—C11—N3	178.21 (16)
N1—Zn1—N3—C11	33.64 (17)	N2—C10—C11—C12	176.96 (15)
Cl3—Zn1—N3—C11	153.42 (11)	C9—C10—C11—C12	−2.6 (3)
Cl2—Zn1—N3—C11	−92.24 (11)	C5—N1—C1—C2	−0.4 (3)
C1—N1—C5—C4	0.1 (2)	Zn1—N1—C1—C2	177.41 (15)
Zn1—N1—C5—C4	−177.91 (13)	C3—C2—C1—N1	0.2 (3)
C1—N1—C5—C6	−178.31 (15)	N3—C15—C14—C13	−0.3 (3)
Zn1—N1—C5—C6	3.69 (18)	C15—C14—C13—C12	0.1 (3)
C3—C4—C5—N1	0.3 (3)	C11—C12—C13—C14	0.2 (3)
C3—C4—C5—C6	178.53 (17)	C1—C2—C3—C4	0.1 (3)
C6—N2—C10—C9	1.4 (2)	C5—C4—C3—C2	−0.4 (3)
Zn1—N2—C10—C9	−168.50 (13)	C9—C8—C7—C6	1.1 (3)
C6—N2—C10—C11	−178.11 (14)	N2—C6—C7—C8	−0.1 (3)
Zn1—N2—C10—C11	11.94 (18)	C5—C6—C7—C8	−178.83 (16)

### Hydrogen-bond geometry (Å, º)

**Table:**

*D*—H···*A*	*D*—H	H···*A*	*D*···*A*	*D*—H···*A*
C4—H4···Cl2^i^	0.93	2.68	3.518 (2)	151
C13—H13···Cl2^ii^	0.93	2.81	3.686 (2)	158

Symmetry codes: (i) −*x*+1, −*y*+1, −*z*; (ii) −*x*, −*y*, −*z*.
