# Supplementary figures and images for: Crystal structure of di­chlorido­(2,2′:6′,2′′-terpyridine-κ3 N,N′,N′′)zinc: a redeter­min­ation
Source: Acta Crystallogr Sect E Struct Rep Online. 2014 Oct 31;70(Pt 11):m382–3. doi: 10.1107/S1600536814023605 (PMC4257341; doi:10.1107/S1600536814023605)

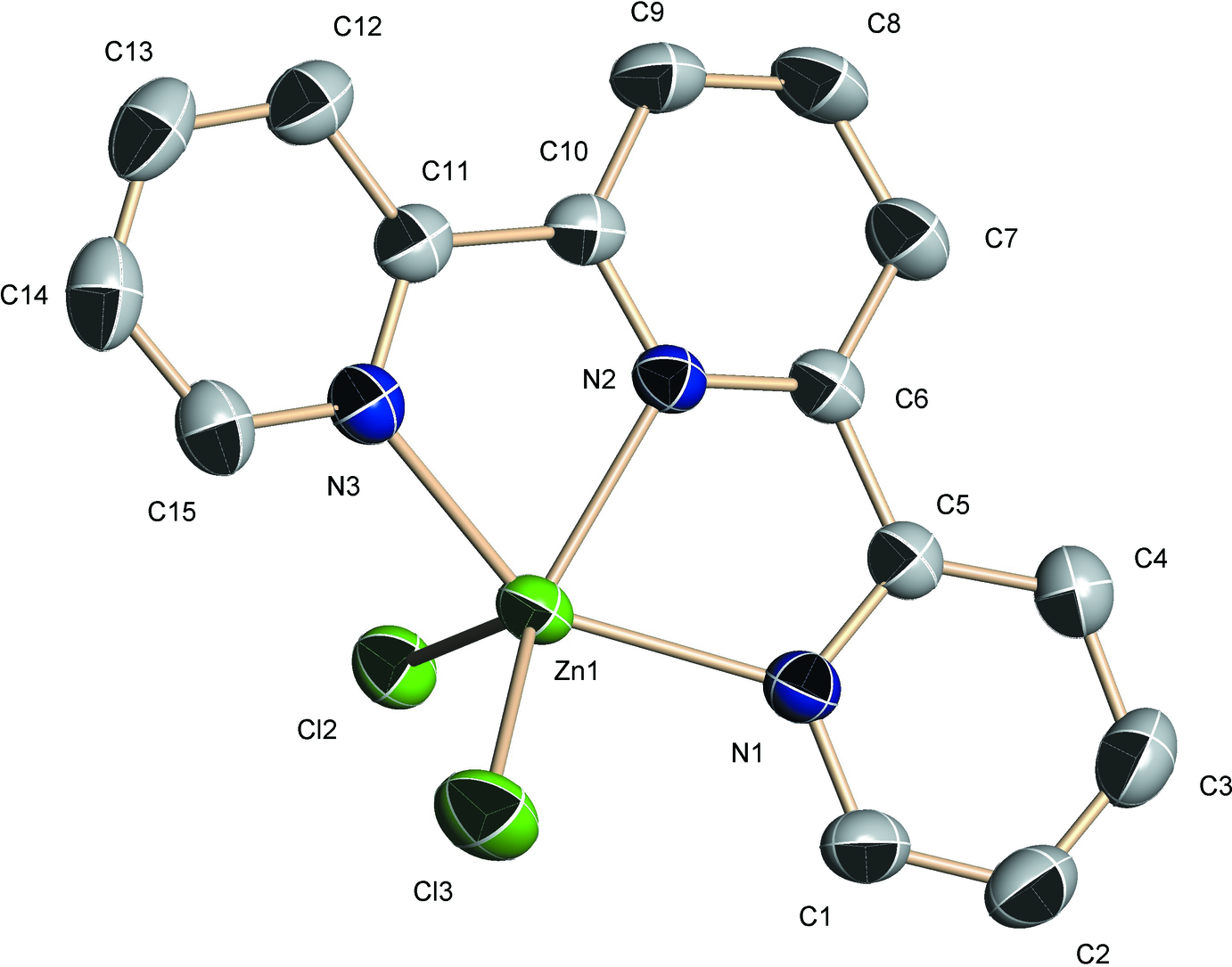

Supplement: Supplementary file 3 [file e-70-0m382-fig1.tif]

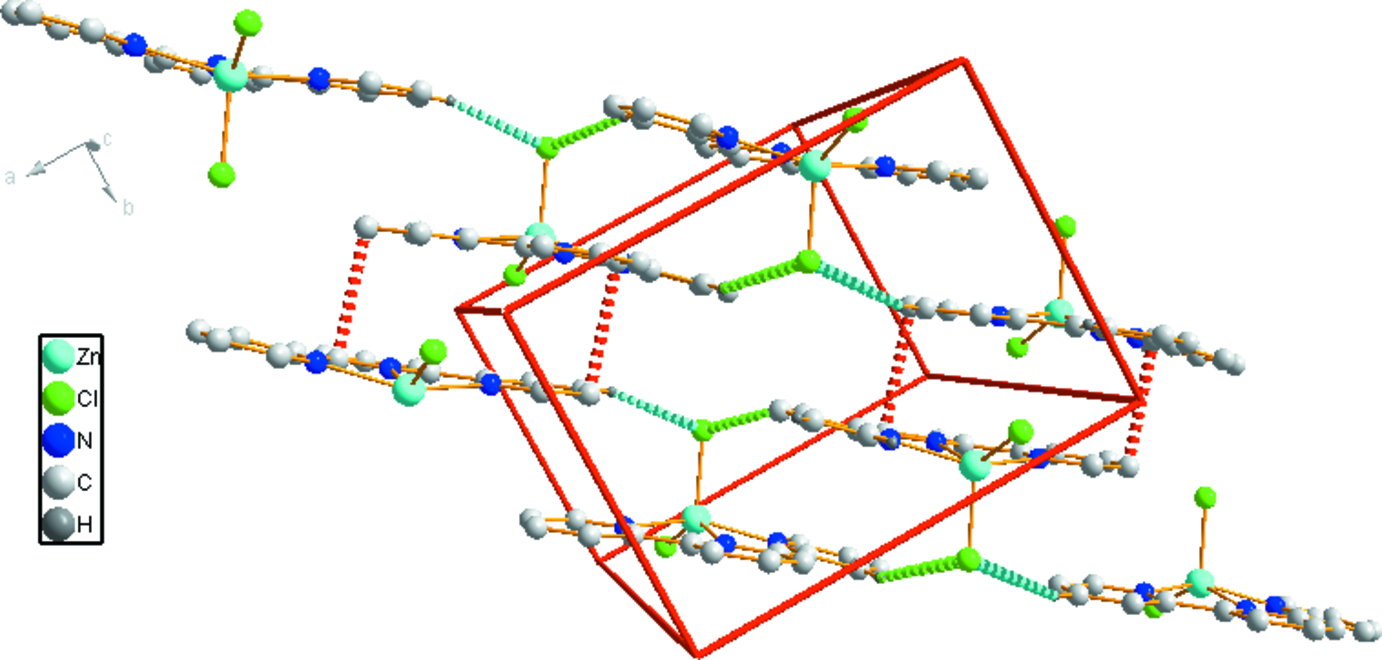

Supplement: Supplementary file 4 [file e-70-0m382-fig2.tif]

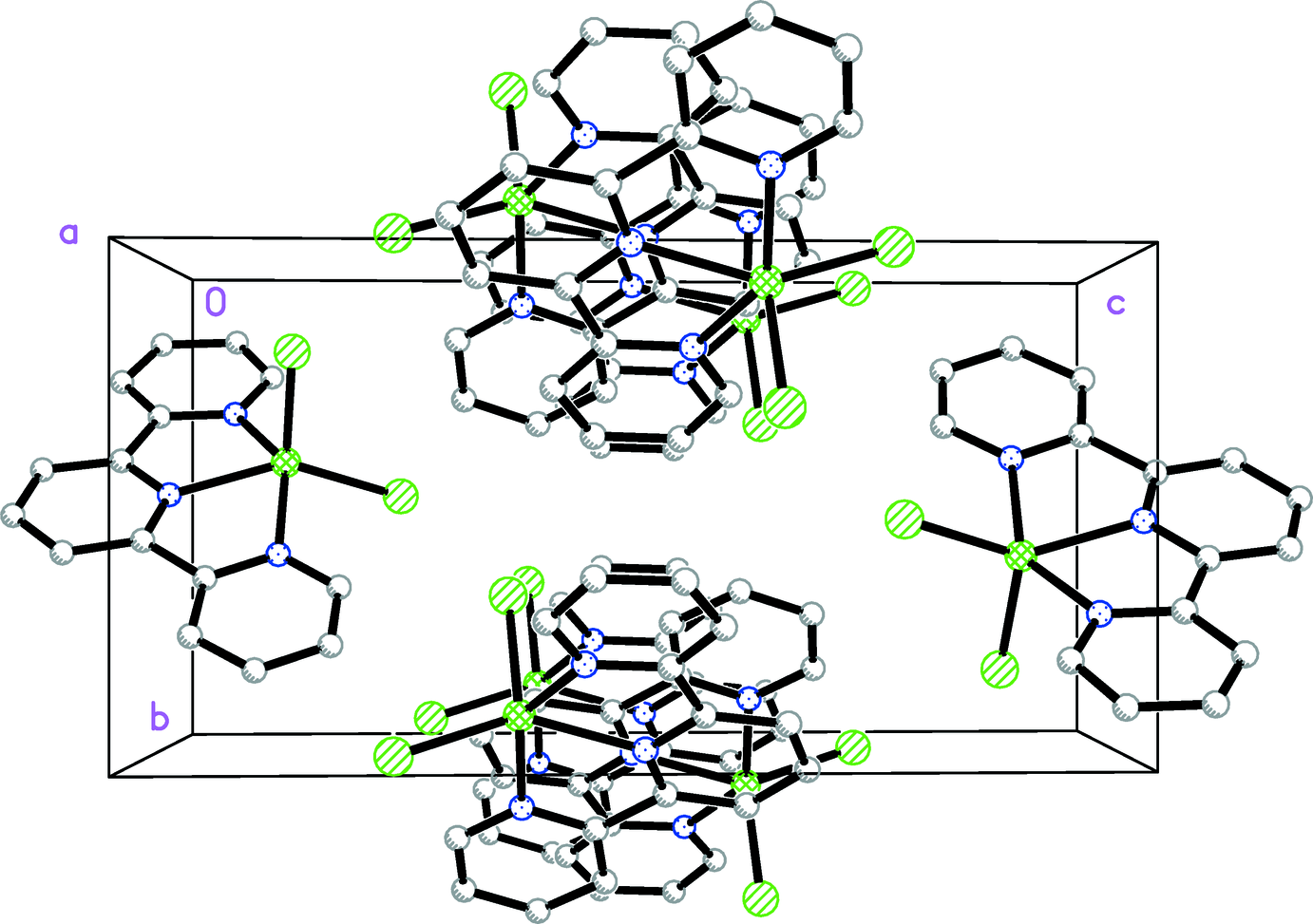

Supplement: Supplementary file 5 [file e-70-0m382-fig3.tif]
